# Chloroplast SNP-marker as powerful tool for differentiation of *Populus* species in reliable poplar breeding and barcoding approaches

**DOI:** 10.1186/1753-6561-5-S7-P56

**Published:** 2011-09-13

**Authors:** Hilke Schroeder, Aki Höltken, Matthias Fladung

**Affiliations:** 1 Johann Heinrich von Thünen Institut (vTI), Institute for Forest Genetics, 22927 Grosshansdorf, Germany; 2 University of Hamburg, Department of Wood Science, Wood Biology, 21031 Hamburg, Germany

## Background

The genus *Populus* is one of the world’s most important tree genera. High growth rates, particularly of some interspecies hybrids, and a broad range of applicability from wood and paper to energy production, led to their widespread cultivation in Europe and North America. For the use in SRCs (short rotation coppices), in particular interspecies-hybrids are well suited because of their superior growth and advanced resistance traits. However, *Populus* hybrids are often morphologically extreme variable and can show either more criteria of the one or the other parental species. Thus, species identification within the genus *Populus* using morphological characters can sometimes be difficult. Furthermore, systematically performed records during breeding or vegetative propagation of poplar hybrids and/or clones are not available to date. For breeding activities with the priority of registration of new high-efficiency clones, clear species identification is inevitably necessary. Therefore, we evaluate the usability of already published plant barcoding regions (“barcoding”; Barcode of Life [http://www.barcodeoflife.org/]) for their efficacy to differentiate seven often used poplar species. We present data on amplification success of the barcoding regions by using the already published primers. Moreover, novel primers were established in promising chloroplast regions to differentiate *Populus* species.

## Methods

Twenty three published barcoding primer combinations were used for PCR amplification of coding and non-coding (intergenic spacers) regions. Additionally, 17 primer combinations have been newly designed taken advantage of the sequence of the *Populus trichocarpa* chloroplast genome. Obtained sequences were aligned and screened for presence of SNPs by using either the software SeqMan 7.1.0 from DNAStar (Lasergene, GATC Biotech, Konstanz, Germany) or Sequencher 4.9 (Gene Codes Corporation, Ann Arbor, USA). The sequences around the SNPs were checked for restriction sites using the software NEBcutter V2.0 from New England BioLabs Inc (Ipswich, USA).

## Results

Twelve of the 23 used barcoding primer combinations and fifteen of the seventeen newly designed primer combinations resulted in PCR amplification products in all individuals for the seven species tested. Twenty four of these amplification products have been sequenced and checked for species-specific SNPs or indels. Three chloroplast regions revealed species-specific SNPs using the original barcoding primers and within seven chloroplast regions species-specific SNPs or indels were identified when using the newly designed primers. A ranking, taking into account the percentage of variable sites for the ten chloroplast regions, reveals the first five places being occupied by intergenic spacers: *trnH-psbA*, *psbK-psbI*, *trnG-psbK*, *ndhE-ndhG* and *rbcL-accD,* followed by two coding regions: *rpoC* and *rbcL*, again one intergenic spacer, *rps2-rpoC2*, and finally two coding regions *rpoB* and *matK*. Besides the usability of the identified SNPs and indels as contribution to the “barcoding of life” project, they should also be used to identify *Populus* species for breeding purposes. For an efficient application, rapid test methods without the need for sequencing or capillary electrophoresis facilities are required. Thus, all sequences with SNPs were checked for restriction sites for use as PCR-RFLPs. For five of the 21 identified SNPs suitable restriction enzymes were found. Thus, we can identify five of the seven used species by using PCR-RFLPs.

## Discussion and conclusions

Altogether, the use of intergenic spacers seems to be more successful to differentiate closely related species as given within the genus *Populus*, because of the higher overall variability. Thus, the choice of suitable barcoding regions is obviously different for different groups of land plants. Our results support the recommendation of some authors of focussing the examinations of the plastid genome on the barcoding potential of the *trnH-psbA* spacer. We recommend focusing even more on intergenic spacers in general (for example *trnG-psbK*). And, therefore, our results also follow the idea of several authors in using multi-locus combinations, because differentiation of only seven species within the genus *Populus* requires three chloroplast regions.

Our study clearly show that SNP markers combined with methods as PCR-RFLPs and length polymorphisms are convenient, easy to use, and - necessary in most breeding programs – fast and of low cost for application in breeding projects.

